# Trends in socioeconomic disparities in a rapid under-five mortality transition: a longitudinal study in the United Republic of Tanzania

**DOI:** 10.2471/BLT.15.154658

**Published:** 2015-02-25

**Authors:** Almamy Malick Kanté, Rose Nathan, Elizabeth F Jackson, Francis Levira, Stéphane Helleringer, Honorati Masanja, James F Phillips

**Affiliations:** a Heilbrunn Department of Population and Family Health, Mailman School of Public Health, Columbia University, 60 Haven Avenue (B2), New York, NY 10032, United States of America (USA).; b Ifakara Health Institute, Dar es Salaam, United Republic of Tanzania.; c Bloomberg School of Public Health, Johns Hopkins University, Baltimore, USA.

## Abstract

**Objective:**

To explore trends in socioeconomic disparities and under-five mortality rates in rural parts of the United Republic of Tanzania between 2000 and 2011.

**Methods:**

We used longitudinal data on births, deaths, migrations, maternal educational attainment and household characteristics from the Ifakara and Rufiji health and demographic surveillance systems. We estimated hazard ratios (HR) for associations between mortality and maternal educational attainment or relative household wealth, using Cox hazard regression models.

**Findings:**

The under-five mortality rate declined in Ifakara from 132.7 deaths per 1000 live births (95% confidence interval, CI: 119.3–147.4) in 2000 to 66.2 (95% CI: 59.0–74.3) in 2011 and in Rufiji from 118.4 deaths per 1000 live births (95% CI: 107.1–130.7) in 2000 to 76.2 (95% CI: 66.7–86.9) in 2011. Combining both sites, in 2000–2001, the risk of dying for children of uneducated mothers was 1.44 (95% CI: 1.08–1.92) higher than for children of mothers who had received education beyond primary school and in 2010–2011, the HR was 1.18 (95% CI: 0.90–1.55). In contrast, mortality disparities between richest and poorest quintiles worsened in Rufiji, from 1.20 (95% CI: 0.99–1.47) in 2000–2001 to 1.48 (95% CI: 1.15–1.89) in 2010–2011, while in Ifakara, disparities narrowed from 1.30 (95% CI: 1.09–1.55) to 1.15 (95% CI: 0.95–1.39) in the same period.

**Conclusion:**

While childhood survival has improved, mortality disparities still persist, suggesting a need for policies and programmes that both reduce child mortality and address socioeconomic disparities.

## Résumé

Objectif** Examiner les tendances en matière de disparités socioéconomiques et de taux de mortalité des moins de cinq ans dans des zones rurales de Tanzanie entre 2000 et 2011.**

Méthodes** Nous avons utilisé des données longitudinales sur les naissances, les décès, les migrations, le niveau d'instruction des mères et les caractéristiques des ménages provenant des systèmes de surveillance sanitaire et démographique d'Ifakara et de Rufiji. Nous avons estimé les ratios de risque (RR) à l’égard des associations entre la mortalité et le niveau d'instruction des mères ou la richesse relative des ménages à l'aide de modèles de régression à risques de Cox.**

Résultats** Le taux de mortalité des moins de cinq ans est passé, à Ifakara, de 132,7 décès pour 1000 naissances vivantes (intervalle de confiance de 95%, IC: 119,3–147,4) en 2000 à 66,2 (IC 95%: 59,0–74,3) en 2011 et, à Rufiji, de 118,4 décès pour 1000 naissances vivantes (IC 95%: 107,1–130,7) en 2000 à 76,2 (IC 95%: 66,7–86,9) en 2011. Sur les deux sites confondus, en 2000–2001, le risque de décès des enfants de mères sans instruction était 1,44 fois plus élevé (IC 95%: 1,08–1,92) que pour les enfants de mères ayant reçu une instruction au-delà de l'école primaire. En 2010–2011, le RR était de 1,18 (IC 95%: 0,90-1,55). En revanche, les disparités de mortalité entre les quintiles les plus riches et les plus pauvres se sont aggravées à Rufiji, passant de 1,20 (IC 95%: 0,99–1,47) en 2000–2001 à 1,48 (IC 95%: 1,15–1,89) en 2010–2011, tandis qu'à Ifakara, ces disparités se sont amenuisées, passant de 1,30 (IC 95%: 1,09–1,55) à 1,15 (IC 95%: 0,95–1,39) sur la même période.**

Conclusion** Si la survie des enfants s'est améliorée, des disparités persistent au niveau de la mortalité, ce qui laisse entendre que des politiques et des programmes visant à réduire la mortalité infantile et à lutter contre les disparités socioéconomiques sont nécessaires.**

## Resumen

**Objetivo:**

Explorar las tendencias en las desigualdades socioeconómicas y las tasas de mortalidad en menores de cinco años en las zonas rurales de la República Unida de Tanzania entre los años 2000 y 2011.

**Métodos:**

Se utilizaron datos longitudinales sobre nacimientos, fallecimientos, migraciones, nivel educativo de las madres y características familiares de los sistemas de control demográfico y de salud de Ifakara y Rufiji. Se estimaron coeficientes de riesgo (HR) para asociaciones entre la mortalidad y el nivel educativo de las madres o la riqueza relativa de cada hogar, utilizando modelos de regresión de Cox de riesgos.

**Resultados:**

La tasa de mortalidad en menores de cinco años descendió en Ifakara de 132,7 fallecimientos por cada 1 000 nacimientos (intervalo de confianza, IC, del 95%: 119,3–147,4) en el año 2000 a 66,2 (IC del 95%: 59,0–74,3) en el año 2011 y en Rufiji de 118,4 fallecimientos por cada 1 000 nacimientos (IC del 95%: 107,1-130,7) en el año 2000 a 76,2 (IC del 95%: 66,7–86,9) en el año 2011. Combinando ambos lugares, entre 2000 y 2001, el riesgo de fallecimiento en niños con madres sin educación fue de 1,44 (IC del 95%: 1,08–1,92) más que en niños con madres que recibieron educación más allá de la escuela primaria. Entre 2010 y 2011, el HR fue de 1,18 (IC del 95%: 0,90–1,55). En cambio, las desigualdades en la mortalidad entre la población más rica y la población más pobre aumentaron en Rufiji, de 1,20 (IC del 95%: 0,99-1,47) en 2000–2001 a 1,48 (IC del 95%: 1,15–1,89) en 2010–2011, mientras que en Ifakara la desigualdad se redujo de 1,30 (IC del 95%: 1,09-1,55) a 1,15 (IC del 95%: 0,95–1,39) durante el mismo periodo.

**Conclusión:**

A pesar de que la supervivencia infantil ha mejorado, las desigualdades en las tasas de mortalidad siguen presentes, lo que sugiere una necesidad de que las políticas y los programas reduzcan la mortalidad infantil y aborden las desigualdades socioeconómicas.

## ملخص

**الغرض:**

استكشاف نزعات الفوارق الاجتماعية والاقتصادية ومعدلات الوفيات للأطفال دون سن الخامسة في المناطق الريفية من جمهورية تنزانيا المتحدة في الفترة ما بين عام 2000 وعام 2011.

**الأسلوب:**

قمنا باستخدام بيانات طولية متعلقة بالمواليد، والوفيات، والهجرات، والتحصيل التعليمي للأمهات، وخصائص الأسرة من أنظمة المراقبة الصحية والديموغرافية في "إفكارا" و"روفيجي". وقمنا بتقدير نسب الخطورة (HR) الخاصة بالارتباطات ما بين الوفيات والتحصيل التعليمي للأمهات أو حجم الثروة النسبي للأسرة، وذلك باستخدام نماذج كوكس لقياس معدل تحوّف المخاطر.

**النتائج:**

انخفض معدل الوفيات للأطفال دون سن الخامسة في "إفكارا" من 132.7 وفيات من بين 1000 مولود على قيد الحياة (بنسبة أرجحية مقدارها 95%: 119.3–147.4) في عام 2000 إلى 66.2 (بنسبة أرجحية مقدارها 95%: 59.0–74.3) في عام 2011 وفي "روفيجي" من 118.4 وفيات من بين 1000 مولود على قيد الحياة (بنسبة أرجحية مقدارها 95‏%: 107.1–130.7) في عام 2000 إلى 76.2 (بنسبة أرجحية مقدارها ‏95%: 66.7–86.9) في عام 2011. وبالجمع بين كلا الموقعين، في الفترة من عام 2000 إلى 2001، بلغ معدل الخطر لوفاة الأطفال من أمهات غير متعلمين 1.44 (بنسبة أرجحية مقدارها 95‏%: 1.08–1.92) أعلى من معدل وفاة الأطفال من أمهات استمروا في تلقي التعليم إلى ما بعد المرحلة الابتدائية. في الفترة من عام 2010 إلى عام 2011، بلغت نسبة الخطورة 1.18 (بنسبة أرجحية مقدارها ‏95%: 0.90–1.55). وفي المقابل تدهورت الفوارق في الوفيات ما بين الشرائح المئوية الأغنى والأفقر في "روفيجي"، من 1.20 (بنسبة أرجحية مقدارها 95‏%: 0.99–1.47) من عام 2000 إلى عام 2001 إلى 1.48 (بنسبة أرجحية مقدارها 95‏%: 1.15–1.89) من عام 2010 إلى عام 2011، بينما ضاقت الفوارق في "إفكارا" من 1.30 (بنسبة أرجحية مقدارها ‏95%: 1.09–1.55) إلى 1.15 (بنسبة أرجحية مقدارها 95‏%: 0.95–1.39) في نفس الفترة.

**الاستنتاج:**

في الوقت الذي تحسنت فيه معدلات بقاء الأطفال على قيد الحياة، فإن الفوارق في معدل الوفيات لا تزال قائمة، مما يشير إلى الحاجة إلى وضع سياسات وبرامج تحد من وفيات الأطفال ومعالجة الفوارق الاجتماعية والاقتصادية على حد سواء.

## 摘要

**目的:**

旨在探索坦桑尼亚联合共和国 2000 年至 2011 年间农村地区社会经济差距和五岁以下儿童死亡率的趋势。

**方法:**

我们利用伊法卡拉和鲁菲吉卫生和人口统计监控系统得出有关出生、死亡、迁移、母亲受教育程度以及家庭特征等纵向数据。 我们利用 Cox 风险回归模型，评估危害比 (HR)，了解死亡和母亲受教育程度或相关家庭财富之间的关系。

**结果:**

伊法卡拉五岁以下儿童死亡率从 2000 年的 132.7‰（95% 置信区间 (CI)： 119.3–147.4) 下降至 2011 年的 66.2‰ (95%CI: 59.0–74.3)，鲁菲吉则从 2000 年的 118.4‰ (95% CI: 107.1–130.7) 下降至 2011 年的 76.2‰ (95% CI: 66.7–86.9)。综合两地数据，2000–2001 年间，母亲未受教育的孩子死亡风险比母亲受过小学以上教育的孩子高出 1.44 (95% CI: 1.08–1.92)。2010–2011 年间，HR 为 1.18 (95% CI: 0.90–1.55). 相对于鲁菲吉最富有和最贫困的五分之一人口之间的死亡差距加大，从 2000–2001 年间的 1.20 (95% CI: 0.99-1.47) 增加至 2010–2011 年间的 1.48 (95% CI: 1.15–1.89)，而在伊法卡拉，差距从 1.30 (95% CI: 1.09–1.55) 缩减至 1.15 (95% CI: 0.95–1.39)。

**结论:**

尽管儿童生存率有所提升，但是死亡差距仍然存在，这表明需要制定政策和计划，来同时降低儿童死亡率和解决社会经济差距。

## Резюме

**Цель:**

Изучить тенденции изменений в социально-экономическом неравенстве и показателях смертности детей в возрасте до пяти лет в сельской местности Объединенной Республики Танзания в период 2000–2011 гг.

**Методы:**

Были использованы многолетние данные о рождениях, смертях, миграции, уровне образования матерей и особенностях семей, полученные из систем медико-санитарного и демографического наблюдения города Ифакара и округа Руфиджи. Для выявления взаимосвязи между уровнем смертности и уровнем образования матерей или относительным благосостоянием семей были подсчитаны отношения рисков (ОР) с помощью регрессионных моделей пропорциональных рисков Кокса.

**Результаты:**

В городе Ифакара уровень смертности детей в возрасте до пяти лет снизился с 132,7 смерти на 1 000 живорожденных детей (95%-й доверительный интервал, ДИ: 119,3–147,4) в 2000 году до 66,2 (95%-й ДИ: 59,0–74,3) в 2011 году, а в округе Руфиджи — с 118,4 смерти на 1 000 живорожденных детей (95%-й ДИ: 107,1–130,7) в 2000 году до 76,2 (95%-й ДИ: 66,7–86,9) в 2011 году. Согласно данным для обоих мест в 2000–2001 годах вероятность смерти для детей, чьи матери не имели образования, в 1,44 раза (95%-й ДИ: 1,08–1,92) превышала вероятность смерти для детей, чьи матери получили образование выше уровня начальной школы. В 2010–2011 годах ОР составляло 1,18 (95%-й ДИ: 0,90–1,55). В то же время в Руфиджи увеличились расхождения в уровнях смертности между самым бедным и самым зажиточным квинтилями: с 1,20 (95%-й ДИ: 0,99–1,47) в 2000–2001 гг. до 1,48 (95%-й ДИ: 1,15–1,89) в 2010–2011 гг., а в г. Ифакара расхождения уменьшились с 1,30 (95%-й ДИ: 1,09–1,55) до 1,15 (95%-й ДИ: 0,95–1,39) в тот же период.

**Вывод:**

Несмотря на улучшение показателей детского выживания, расхождения в уровнях смертности по-прежнему сохраняются, что говорит о необходимости политических действий и программ, нацеленных на снижение детской смертности и устранение социально-экономического неравенства.

## Introduction

Despite evidence that social, demographic and residential disparities in the mortality of children younger than 5 years have declined, in many low-income countries – especially in sub-Saharan Africa – such disparities still exist.^1^ Measures of socioeconomic status such as poverty,^2^^–^^7^ maternal educational attainment,^5^^,^^8^^–^^10^ social class,^11^^,^^12^ geographical setting,^13^^,^^14^ and rural and remote residence^15^^,^^16^ have all been shown to be associated with under-five mortality rates. However, less is known about whether socioeconomic disparities decrease as a result of a reduction in child mortality.

In the United Republic of Tanzania, the under-five mortality rate has declined by 40% from 137 deaths per 1000 live births in 1992–1996 to 81 in 2006–2010.^17^^–^^20^ However, there are still disparities in risk of death among children younger than 5 years. Mortality is highest among infants, boys, children of uneducated mothers, children of youngest or oldest mothers and children from relatively poor households.

Here we describe the mortality transition between 2000 and 2011 in three rural districts of the United Republic of Tanzania and show the longitudinal associations with indicators of health equity, i.e. maternal and household factors.

## Methods

### Surveillance system characteristic

We used data from the Ifakara Health Institute’s integrated health and demographic surveillance system that continuously collected data on mortality levels in conjunction with data on social and economic factors.^21^^,^^22^ The data cover the entire population of the surveillance sites, their household relationships and demographic events such as births, deaths and migration in and out of households.

The surveillance system collects data in three rural districts in the United Republic of Tanzania. The Rufiji surveillance system is located in Rufiji district in the eastern part of the country while the Ifakara surveillance system is located in Kilombero and Ulanga districts in the south central part of the country. The surveillance sites cover approximately 4200 km^2^ (1800 in Rufiji and 2400 in Ifakara) and 58 villages (33 in Rufiji and 25 in Ifakara). The sites are largely rural with economies that are dominated by subsistence farming, fishing and petty trading.^21^^,^^22^

Baseline census data were obtained in September 1996 for the Ifakara surveillance population and in November 1998 for the Rufiji surveillance population. Since then, the data have been updated in 120-day intervals by interviewers revisiting households to register the occurrence of all births, deaths, household migrations and marital status changes. Pregnancies are documented and pregnancy outcomes are solicited in subsequent household visits. In annual cycles, data on socioeconomic indicators are collected such as level of education, occupation and household socioeconomic status measured with proxy markers – such as household possessions, household water and sanitation and building materials.

In the beginning of 2011, the total surveillance population was about 190 000 persons (82 012 persons in Rufiji and 108 655 persons in Ifakara), of which 22 981 (12.1%) were younger than 5 years. Major causes of mortality for these children included malaria and fever, pneumonia, prematurity and low birth weight, birth injuries, asphyxia, anaemia and malnutrition.^23^

### Data Analysis

#### Variable definitions

We collected information on all children younger than 5 years living in the surveillance sites between 1 January 2000 and 31 December 2011. Beginning with all children younger than 5 years on 1 January 2000, we added children who entered the surveillance system through birth or migration into the study area. Children were observed until the end of the study period, 31 December 2011, unless they were censored due to death, migration out of the study area or reaching the age of 5 years.

Variables included sex of child, birth order (first, second, third and fourth or higher), maternal age at birth of child (younger than 20, 20–34 and older than 35 years), maternal educational attainment (none, incomplete primary level, complete primary level and secondary level or more) and household wealth quintile (first, second, third, fourth and fifth, where first represent the poorest and the fifth the richest). Time period was included into the analysis as a continuous variable from 2000 to 2011 to account for changes in mortality over time.

#### Classifying relative wealth

We constructed an asset index as a proxy for wealth using the principal components analysis (PCA).^24^ PCA has been shown to be a reliable method for classifying relative socioeconomic status in rural households in developing settings.^25^^,^^26^ Information used to create the assets index included ownership of means of mobility, possession of electronic and electric devices, ownership of household equipment, landholding, livestock, household construction, and type of energy used for cooking or lighting.

### Data limitations

Household socioeconomic data were collected each year in Ifakara from 1997. In Rufiji however, data were collected in 2000, 2004 and in every year since 2007. For the years lacking these data, we used the most recent estimate available. This limitation to our data involved the assumption that the most recent assessment of household socioeconomic status was applicable to subsequent periods and therefore, that household socioeconomic status did not change between 2000 and 2002, between 2003 and 2005 and between 2006 and 2007.

We did not control for possible confounding factors related to child characteristics, such as birth type (single or multiple), or maternal characteristics, such as marital status changes, presence or absence of household heads or the geographic remoteness of households because these data were only collected from 2003 onwards.

#### Regression models

The overall trends in under-five mortality for the period 2000–2011 were calculated using Kaplan–Meier analysis.^27^ We used the Cox proportional hazard model^28^ to test for associations between the risk of child death and characteristics of children, mothers and households with multivariate controls. The efron method was used to handle tied failures.^29^

A multivariate model of child survival was created to measure the relationship between mortality and maternal, child and household characteristics. This multivariate hazard regression model (model 1) was constructed as follows:
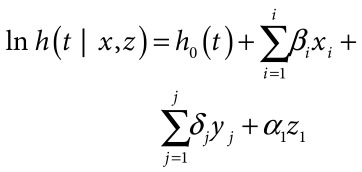
(1)where *h* defines the hazard of childhood death for exact ages in days from *t* = 0 to 60 months and *x* defines *i* indicators of maternal or child characteristics that are unrelated to equity and *y* defines *j* indicators of social and economic disparity and *z* defines time in periods of two calendar years each.

To determine whether mortality disparities by wealth quintiles and educational status were changing during the mortality transition, a model was created that assessed for interactions between time and maternal educational attainment and between time and household wealth. This multivariate hazard regression model (model 2) was constructed as follows:
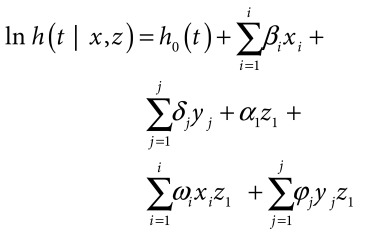
(2)for both model 1 and model 2, three analyses were conducted, one using the Ifakara data set, one using the Rufiji data set and a third using the combined data set. For each main effects variable coefficient, a *P*-value of 0.05 was defined as statistically significant. For model 2, because of low statistical power for interaction tests,^30^^–^^32^ we assessed the interaction coefficients with an error rate of 20% rather than the traditional 5%.^32^ For statistical analyses, we used Stata version 13.1 (StataCorp. LP, College Station, United States of America).

### Ethical statement

Following an explanation of the health and demographic surveillance system, each participant in the surveillance system is asked to provide written informed consent (parents, guardians or next of kin, on behalf of all minors/children enrolled in our study). Local leaders at village and district levels were informed about the survey and the use of data. Respondents remained anonymous. The data collection procedure was reviewed and approved by the ethical review committees of the Ministry of Health and Social Welfare of the United Republic of Tanzania, the National Medical Research Coordination Committee of the National Institute for Medical Research and the Ifakara Health Institute’s Institutional Review Board. We sought authorization for use of data from the Ifakara Health Institute through the Connect project (IHI/IRB/No.16–2010). Ethical clearance for the analysis was accorded by the Columbia University Medical Center Institutional Review Board (Protocol-AAAF3452).

## Results

### Characteristics

During the period 2000–2011, 140 162 children younger than 5 years were resident in study sites, of which 55.7% (78 136) was registered in Ifakara and 44.3% (62 026) in Rufiji. They contributed to a combined 325 960 person-years of observation. Half of the children were boys, (39 034 boys in Ifakara and 30 919 boys in Rufiji). Forty-seven percent (28 861) of mothers in Rufiji and 35.2% (27 472) in Ifakara did not have any formal education. Of the 83 929 mothers who were educated, 14.6% (11 412 in Ifakara and 9074 in Rufiji) had not completed the primary level of education. In Ifakara, 47.8% (37 349) of the mothers had completed the primary level; corresponding percentage was 35.0% (21 689) in Rufiji. Only 2.4% (1903) of mothers in Ifakara and 3.9% (2402) in Rufiji acquired schooling beyond the secondary level. There were 14 828 children living in the poorest quintile of households in Ifakara and 10 256 in Rufiji (Table 1).

**Table 1:** Characteristics of the study population in Ifakara and Rufiji Health and Demographic Surveillance Systems, United Republic of Tanzania, 2001–2011

Variable	No. (%)		
	All children	Ifakara	Rufiji
**Total**	140 162 (100)	78 136 (55.7)	62 026 (44.3)
**Child**			
Sex			
Boy	69 953 (49.9)	39 034 (50.0)	30 919 (49.9)
Girl	70 209 (50.1)	39 102 (50.0)	31 107 (50.1)
Birth order			
First	51 754 (36.9)	30 970 (39.6)	20 784 (33.5)
Second or third	49 134 (35.1)	27 826 (35.6)	21 308 (34.4)
Fourth or more	39 274 (28.0)	19 340 (24.8)	19 934 (32.1)
**Mother**			
Age group, years			
< 20	38 464 (27.4)	21 782 (27.9)	16 682 (26.9)
20–34	83 765 (59.8)	47 390 (60.7)	36 375 (58.7)
≥ 35	17 933 (12.8)	8 964 (11.5)	8 969 (14.5)
Education			
No education	56 333 (40.2)	27 472 (35.2)	28 861 (46.5)
Primary incomplete	20 486 (14.6)	11 412 (14.6)	9 074 (14.6)
Primary complete	59 038 (42.1)	37 349 (47.8)	21 689 (35.0)
Secondary or more	4 305 (3.1)	1 903 (2.4)	2 402 (3.9)
**Household**			
Wealth quintile^a^			
First	25 084 (19.3)	14 828 (20.4)	10 256 (17.9)
Second	25 526 (19.6)	14 545 (20.0)	10 981 (19.2)
Third	28 299 (21.8)	15 635 (21.5)	12 664 (22.1)
Fourth	26 970 (20.8)	14 320 (19.7)	12 650 (22.1)
Fifth	24 125 (18.6)	13 419 (18.5)	10 706 (18.7)

^a^ First quintile represents the poorest households and fifth quintile represents the richest. Overall, 6.9% of households in Ifakara and 7.7% in Rufiji have missing data. These percentages varied by year. In 2000, 8.0% of households in Ifakara had missing data and 15.8% in Rufiji. In 2011, it was 9.4% in Ifakara and 7.1% in Rufiji.

Note: Inconsistencies arise in some values due to rounding. Ifakara Health and Demographic Surveillance System covers part of Kilombero and Ulanga districts in Morogoro region and Rufiji Health and Demographic Surveillance System covers the half of Rufiji district in Pwani region.

### Trends

**Fig. 1:**
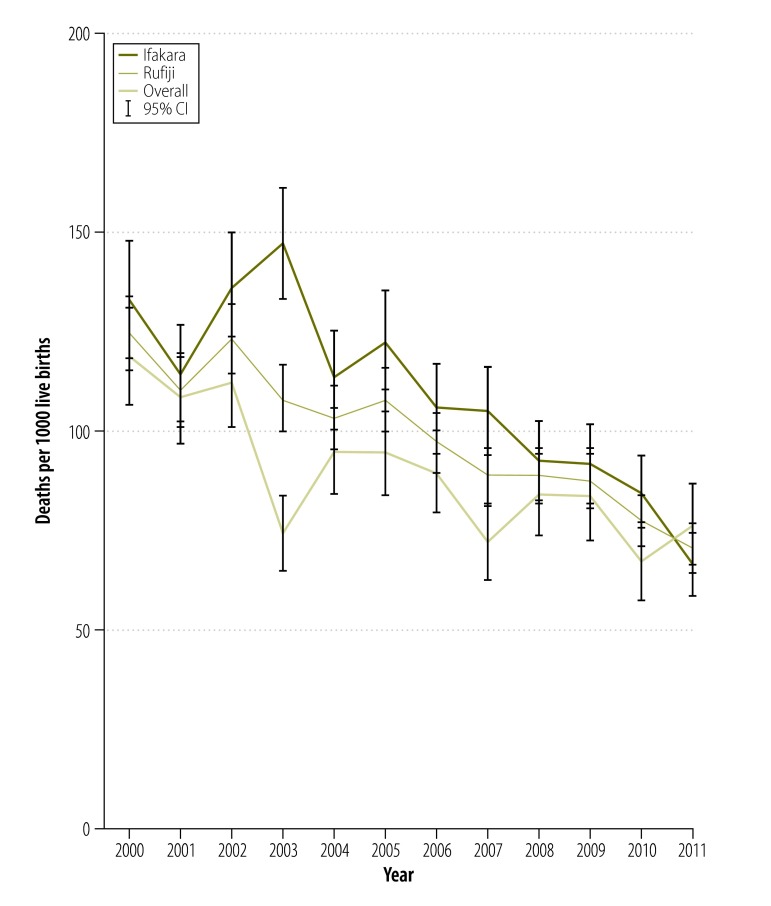
Under-five mortality trends in Ifakara and Rufiji, United Republic of Tanzania, 2000–2011

Note: Trends were calculated from the Kaplan–Meier survival function as the probability of dying between birth and the fifth birthday (5q0).

The under-five mortality rate, measured as a probability of dying before 5 years of age, declined by 43.4%, from 124.4 deaths per 1000 live births in 2000 (95% confidence interval, CI: 115.7–133.7) to 70.4 in 2011 (95% CI: 64.5–76.7). During this period, the reduction was greater in Ifakara – from 132.7 deaths per 1000 live births (95% CI: 119.3–147.4) to 66.2 (95% CI: 59.0–74.3) – than Rufiji – from 118.4 deaths per 1000 live births (95% CI: 107.1–130.7) to 76.2 (95% CI: 66.7–86.9). There was a slight increase in deaths between 2010 and 2011 (Fig. 1).

### Regression models

#### Model 1

Boys, children of low birth order, children of youngest or oldest mothers, children of uneducated mothers and mothers who had only attended primary school and children of poor households had a statistically significant higher risk of dying compared to others. Overall, children living in the poorest quintiles of households had a risk of dying that was 1.28 (95% CI: 1.18–1.38) times greater than children living in the richest households. Children of uneducated mothers had a risk of dying that was 1.31 (95% CI: 1.12–1.54) times greater than children whose mothers had received education beyond primary school. Over the course of the study period, the risk of dying was significantly reduced by more than one-third in Ifakara and in Rufiji. Overall, in 2010–2011 the risk of dying was 0.65 (95% CI: 0.60–0.71) times lower than in 2000–2001 (Table 2).

**Table 2:** Characteristics of child, mother and household associated with the risk of dying under the age of five years in Ifakara and Rufiji, United Republic of Tanzania, 2000–2011

Variable	HR^a^ (95% CI)		
	Ifakara^b^	Rufiji^c^	All^d^
**Child**			
Sex			
Boy	1.08 (1.01–1.15)	1.08 (1.01–1.16)	1.08 (1.03–1.14)
Girl	Reference	Reference	Reference
Birth order			
First	1.38 (1.25–1.54)	1.34 (1.19–1.52)	1.40 (1.29–1.52)
Second or third	Reference	Reference	Reference
Fourth or more	1.18 (1.08–1.29)	1.11 (1.01–1.22)	1.17 (1.09–1.24)
**Mother**			
Age group, years			
< 20	1.07 (0.98–1.18)	1.04 (0.93–1.16)	1.04 (0.97–1.12)
20–34	Reference	Reference	Reference
≥ 35	1.10 (0.99–1.23)	1.16 (1.04–1.30)	1.13 (1.05–1.22)
Education			
No education	1.34 (1.05–1.70)	1.30 (1.05–1.61)	1.31 (1.12–1.54)
Primary incomplete	1.56 (1.23–1.99)	1.28 (1.02–1.60)	1.49 (1.27–1.75)
Primary complete	1.27 (1.01–1.60)	1.15 (0.92–1.42)	1.27 (1.08–1.48)
Secondary or more	Reference	Reference	Reference
**Household**			
Wealth quintile^e^			
First	1.21 (1.08–1.34)	1.32 (1.17–1.49)	1.28 (1.18–1.38)
Second	1.06 (0.95–1.18)	1.18 (1.04–1.33)	1.11 (1.03–1.21)
Third	1.01 (0.91–1.12)	1.08 (0.96–1.22)	1.05 (0.97–1.14)
Fourth	1.04 (0.93–1.16)	1.13 (1.00–1.27)	1.07 (0.99–1.16)
Fifth	Reference	Reference	Reference
**Period^f^**			
2000–2001	Reference	Reference	Reference
2002–2003	1.17 (1.05–1.31)	0.85 (0.76–0.95)	1.01 (0.93–1.09)
2004–2005	0.99 (0.88–1.10)	0.86 (0.76–0.96)	0.92 (0.85–1.00)
2006–2007	0.89 (0.80–1.00)	0.72 (0.64–0.81)	0.81 (0.75–0.88)
2008–2009	0.78 (0.69–0.87)	0.77 (0.68–0.86)	0.78 (0.72–0.84)
2010–2011	0.63 (0.56–0.71)	0.66 (0.58–0.76)	0.65 (0.60–0.71)

CI: confidence interval; HR: hazard ratio.

^a^ HRs were calculated using multivariate analysis and Cox model.

^b^ In the analysis 73 047 children and 3744 events were included.

^c^ In the analysis 58 366 children and 3019 events were included.

^d^ In the analysis 131 413 children and 6763 events were included.

^e^ First quintile represents the poorest households and fifth quintile represents the richest.

^f^ We used two-year period averages to stabilize rates differentials due to fewer numbers of child deaths in some years.

#### Model 2

Mortality disparities according to maternal educational attainment slightly decreased over time at both surveillance sites. However, reduction in the risk of dying over time for children of uneducated mothers compared to children of mothers who had received education beyond a primary school was not significant in Ifakara and Rufiji, HR: 0.97 (95% CI: 0.84–1.11) and HR: 0.93 (95% CI: 0.83–1.05), respectively (Table 3).

**Table 3:** The interaction between under-five mortality transition and maternal educational attainment or household wealth in Ifakara and Rufiji, United Republic of Tanzania, 2000–2011

Variable	HR (95% CI)^a^		
	Ifakara^b^	Rufiji^c^	All^d^
**Mother’s education**			
No education	1.50 (0.84–2.67)	1.63 (1.01–2.63)	1.50 (1.04–2.16)
Primary incomplete	1.29 (0.97–1.72)	1.26 (0.98–1.62)	1.32 (1.09–1.59)
Primary complete	1.12 (0.93–1.36)	1.09 (0.93–1.28)	1.13 (1.00–1.27)
Secondary or more	Reference	Reference	Reference
**Household’s wealth, quintile^e^**			
First	1.33 (1.06–1.67)	1.16 (0.89–1.50)	1.27 (1.07–1.50)
Second	1.15 (1.02–1.29)	0.96 (0.84–1.10)	1.06 (0.97–1.16)
Third	1.03 (0.95–1.11)	0.92 (0.84–1.00)	0.98 (0.93–1.04)
Fourth	1.01 (0.95–1.07)	0.98 (0.91–1.04)	0.99 (0.95–1.04)
Fifth	Reference	Reference	Reference
**Time period^f^**	0.95 (0.83–1.09)	0.92 (0.82–1.04)	0.94 (0.86–1.03)
Wealth of household*Period			
First*Period	0.98 (0.92–1.04)	1.04 (0.97–1.12)	1.00 (0.96–1.05)
Second* Period	0.97 (0.94–1.00)	1.04 (1.00–1.08)	1.00 (0.98–1.02)
Third* Period	0.99 (0.97–1.01)	1.04 (1.01–1.06)	1.01 (1.00–1.03)
Fourth* Period	1.00 (0.98–1.01)	1.02 (1.00–1.04)	1.01 (1.00–1.02)
Fifth* Period	Reference	Reference	Reference
Mother’s education*Period			
No education* Period	0.97 (0.84–1.11)	0.93 (0.83–1.06)	0.96 (0.88–1.05)
Primary incomplete* Period	0.99 (0.92–1.07)	0.97 (0.91–1.03)	0.98 (0.93–1.03)
Primary complete* Period	0.99 (0.94–1.03)	0.99 (0.95–1.03)	0.99 (0.96–1.02)
Secondary or more* Period	Reference	Reference	Reference

CI: confidence interval; HR: hazard ratio.

^a^ HRs were calculated using multivariate analysis with interaction between time and mother’s education attainment or household’s socioeconomic status, Cox model.

^b^ In the analysis 73 047 children and 3744 events were included.

^c^ In the analysis 58 366 children and 3019 events were included.

^d^ In the analysis 131 413 children and 6763 events were included.

^e^ First quintile represents the poorest households and fifth quintile represents the richest.

^f ^We used two-year period averages to stabilize rates differentials due to fewer numbers of child deaths in some years.

Note: We used Cox proportional hazard analysis with interaction between time and mother’s education attainment or household’s socioeconomic status.

Disparities in mortality due to household wealth showed opposite patterns between the two surveillance sites. In Ifakara, mortality disparities between children living in the richest households and those living in poorer quintiles of households slightly declined over time. Only children living in households that belonged to the second quintile showed a statistically significant reduction over time (HR: 0.97; 95% CI: 0.94–1.00). In Rufiji, wealth-based disparities in mortality widened over time. There was a significant increase between children living in the richest households and those living in households belonging to the second, third and fourth quintiles. Over time, the risk of dying for children living in the second quintile was 1.04 times (95% CI: 1.00–1.08) that of children living in the fifth quintile, for example (Table 3).

Table 4 and Table 5 (available at: http://www.who.int/bulletin/volumes/94/4/15-154658). show how mortality disparities have been fluctuating over time. In 2000–2001, in Ifakara, the risk of dying for children of uneducated mothers was 1.44 (95% CI: 0.92–2.27) higher than for children of mothers who had received education beyond a primary school. In Rufiji, this risk was 1.52 (95% CI: 1.05–2.21). In 2010–2011 this risk had decreased to 1.21 (95% CI: 0.81–1.82) in Ifakara and 1.08 (95% CI: 0.74–1.58) in Rufiji.

**Table 4:** Hazard ratios comparing under-5 mortality and maternal education attainment, by 2-year period, in Rufiji and Ifakara, United Republic of Tanzania, 2000–2011

Period	Maternal educational attainment, HR (95% CI)		
	No education vs secondary or more	Primary incomplete vs secondary or more	Primary complete vs secondary or more
**Ifakara**			
2000–2001	1.44 (0.92–2.27)	1.28 (1.02–1.60)	1.11 (0.96–1.29)
2002–2003	1.39 (0.99–1.96)	1.27 (1.07–1.50)	1.10 (0.99–1.23)
2004–2005	1.35 (1.04–1.75)	1.26 (1.11–1.43)	1.09 (1.00–1.19)
2006–2007	1.30 (1.02–1.66)	1.25 (1.11–1.41)	1.08 (1.00–1.17)
2008–2009	1.25 (0.93–1.70)	1.24 (1.06–1.45)	1.07 (0.97–1.18)
2010–2011	1.21 (0.81–1.82)	1.23 (1.00–1.52)	1.06 (0.92–1.21)
**Rufiji**			
2000–2001	1.52 (1.05–2.21)	1.22 (1.00–1.48)	1.08 (0.95–1.22)
2002–2003	1.42 (1.07–1.88)	1.18 (1.02–1.37)	1.06 (0.97–1.17)
2004–2005	1.33 (1.06–1.66)	1.14 (1.01–1.28)	1.05 (0.98–1.13)
2006–2007	1.24 (0.99–1.55)	1.10 (0.98–1.24)	1.04 (0.96–1.12)
2008–2009	1.16 (0.87–1.54)	1.07 (0.92–1.24)	1.03 (0.93–1.13)
2010–2011	1.08 (0.74–1.58)	1.03 (0.84–1.26)	1.01 (0.89–1.15)
**All**			
2000–2001	1.44 (1.08–1.92)	1.29 (1.11–1.49)	1.12 (1.01–1.23)
2002–2003	1.38 (1.11–1.72)	1.26 (1.13–1.41)	1.10 (1.03–1.18)
2004–2005	1.33 (1.12–1.57)	1.23 (1.13–1.34)	1.09 (1.03–1.15)
2006–2007	1.28 (1.08–1.51)	1.20 (1.11–1.31)	1.08 (1.02–1.13)
2008–2009	1.23 (1.00–1.51)	1.18 (1.06–1.31)	1.06 (0.99–1.14)
2010–2011	1.18 (0.90–1.55)	1.15 (1.00–1.33)	1.05 (0.96–1.15)

CI: confidence interval; HR: Hazard ratio.

**Table 5:** Hazard ratios comparing under-5 mortality and household wealth, in Rufiji and Ifakara, United Republic of Tanzania, 2000–2011

Period	Household’s wealth quintile, HR (95% CI)			
	First vs fifth	Second vs fifth	Third vs fifth	Fourth vs fifth
**Ifakara**				
2000–2001	1.30 (1.09–1.55)	1.11 (1.01–1.22)	1.02 (0.96–1.08)	1.01 (0.96–1.06)
2002–2003	1.27 (1.11–1.44)	1.08 (1.01–1.15)	1.02 (0.97–1.06)	1.01 (0.97–1.04)
2004–2005	1.24 (1.11–1.37)	1.04 (0.99–1.10)	1.01 (0.97–1.04)	1.01 (0.98–1.04)
2006–2007	1.21 (1.08–1.35)	1.01 (0.96–1.07)	1.00 (0.96–1.04)	1.01 (0.98–1.04)
2008–2009	1.18 (1.02–1.36)	0.98 (0.91–1.05)	0.99 (0.95–1.04)	1.01 (0.97–1.04)
2010–2011	1.15 (0.95–1.39)	0.95 (0.86–1.05)	0.99 (0.93–1.05)	1.01 (0.96–1.06)
**Rufiji**				
2000–2001	1.20 (0.99–1.47)	1.00 (0.90–1.10)	0.95 (0.89–1.01)	0.99 (0.94–1.04)
2002–2003	1.25 (1.08–1.45)	1.04 (0.96–1.12)	0.98 (0.93–1.03)	1.01 (0.97–1.05)
2004–2005	1.31 (1.16–1.48)	1.08 (1.01–1.15)	1.02 (0.98–1.06)	1.03 (1.00–1.06)
2006–2007	1.36 (1.18–1.56)	1.12 (1.05–1.20)	1.05 (1.01–1.10)	1.05 (1.01–1.08)
2008–2009	1.42 (1.18–1.70)	1.16 (1.06–1.27)	1.09 (1.03–1.16)	1.06 (1.02–1.11)
2010–2011	1.48 (1.15–1.89)	1.21 (1.07–1.36)	1.13 (1.05–1.22)	1.08 (1.02–1.15)
**All**				
2000–2001	1.27 (1.12–1.45)	1.06 (0.99–1.13)	0.99 (0.95–1.04)	1.00 (0.97–1.03)
2002–2003	1.28 (1.16–1.41)	1.06 (1.01–1.11)	1.00 (0.97–1.04)	1.01 (0.98–1.03)
2004–2005	1.28 (1.19–1.39)	1.06 (1.01–1.10)	1.01 (0.99–1.04)	1.02 (0.99–1.04)
2006–2007	1.29 (1.18–1.40)	1.06 (1.01–1.10)	1.02 (1.00–1.05)	1.02 (1.00–1.04)
2008–2009	1.29 (1.15–1.45)	1.06 (1.00–1.12)	1.04 (1.00–1.08)	1.03 (1.00–1.06)
2010–2011	1.30 (1.12–1.51)	1.06 (0.98–1.14)	1.05 (1.00–1.10)	1.04 (1.00–1.08)

CI: confidence interval; HR: Hazard ratio.

Note: First quintile represents the poorest and the fifth quintile represents the richest.

Results for household wealth showed that over time mortality disparities between the richest and poorer quintiles worsened in Rufiji while in Ifakara, disparities narrowed slightly. In Rufiji, in 2000–2001 the mortality HR for children living in the poorest households was 1.20 (95% CI: 0.99–1.47) times higher than for those living in the richest households. In 2010–2011, the HR had increased to 1.48 (95% CI: 1.15–1.89). In Ifakara, such disparities decreased from 1.30 (95% CI: 1.09–1.55) in 2000–2001 to 1.15 (95% CI: 0.95–1.39) in 2010–2011.

## Discussion

Many developing countries are currently undergoing rapid demographic, economic and under-five mortality transitions. In these countries, research on socioeconomic health inequalities usually focuses only on childhood mortality and its determinants.^33^ Few studies have explored trends in social and economic disparities associated with rapid mortality decline in sub-Saharan Africa.

Here we show a decline in the under-five mortality rate between 2001 and 2011 in Rufiji and Ifakara surveillance sites. However, variables such as the sex of the child, maternal age and educational attainment and the relative household wealth are still associated with an increased risk. During the under-five mortality transition, indicators of social and economic disparities have not narrowed. Thus, while our research confirms other studies in the improvement in childhood mortality, we demonstrate that equity problems remain despite this progress.^34^^–^^36^ It has been shown that there is a differential improvement in proximate mortality determinants across socioeconomic groups, with slower and later improvements among more disadvantaged groups.^37^

National estimates in the United Republic of Tanzania have also shown declines in under-five mortality rates during 2001 and 2011, particularly after 2005 and most prominently in rural areas.^20^ This decline in rural areas is probably related to health system improvement and other factors, such as increased coverage of immunization, of insecticide-treated nets and access to clean and safe water.^38^^–^^41^ Since 2000, public expenditure on health has increased twofold in the country. More resources may enable district health teams to allocate funds for cost–effective interventions in line with the local burden of diseases.^42^ The WHO Integrated Management of Childhood Illness approach, vitamin A supplementation, immunization and insecticide-treated nets were introduced and scaled up during this period.^38^

Our findings on the associations between disparities in the under-five mortality rate and social and demographic characteristics of children and their mothers are consistent with previous studies in sub-Saharan Africa.^6^^,^^8^^,^^10^^,^^25^^,^^43^ It has been demonstrated that in sub-Saharan Africa, boys have a higher risk of dying compared to girls.^44^^–^^46^ Higher maternal educational attainment has been shown to be associated with lower under-five mortality rates at a national level in the United Republic of Tanzania^20^ and elsewhere.^8^^,^^10^ Our findings that childhood mortality disparities by maternal educational attainment narrowed slightly over time are consistent with findings from south Asia.^47^^,^^48^

In rural areas of the United Republic of Tanzania^43^^,^^49^ and other low- and middle-income countries^3^^,^^4^ household wealth is a predictive factor of disparities in under-five mortality. During our study period, mortality disparities between the poorest and richest worsened in Rufiji and remained stable in Ifakara. Therefore, improved child survival does not necessarily indicate improved equity. These findings are consistent with previous studies from Malawi,^34^ Bangladesh^35^ and Chile^36^ but inconsistent with an Indonesian study showing that socioeconomic inequities in childhood mortality declined during a period of economic growth and improved child survival.^47^

In conclusion, our findings suggest that children from all socioeconomic strata are benefitting from improved child survival. We also provide evidence that mortality disparities by maternal educational attainment and household wealth have not significantly narrowed. These results suggest that the longstanding equity problems of rural areas of the country still persist. Policies and health programmes are needed to reduce mortality, and offset social and economic disparities between population subgroups.
